# Linking Cooperative Learning and Emotional Intelligence in Physical Education: Transition across School Stages

**DOI:** 10.3390/ijerph17145090

**Published:** 2020-07-15

**Authors:** Sergio Rivera-Pérez, Benito León-del-Barco, Javier Fernandez-Rio, Jerónimo J. González-Bernal, Damián Iglesias Gallego

**Affiliations:** 1 Physical Education & Exercise Lab, Teacher Training College, University of Extremadura, 10003 Cáceres, Spain; sergioriveraperez@gmail.com; 2 Department of Psychology, Teacher Training College, University of Extremadura, 10003 Cáceres, Spain; bleon@unex.es; 3 Department of Education Sciences, Faculty of Teacher Training and Education, University of Oviedo, 33003 Oviedo, Spain; javier.rio@uniovi.es; 4 Department of Psychology, University of Burgos, 09001 Burgos, Spain; jejavier@ubu.es

**Keywords:** cooperative learning, emotional intelligence, physical education, school stages

## Abstract

The present research had two complementary aims: (a) to examine the associations between cooperative learning (CL) and emotional intelligence (EI) in physical education (PE) and (b) to explore and compare the use of CL in PE in primary education (PED), secondary education (SED) and baccalaureate (BA). A total of 1332 students (682 males, 650 females) took part in the study. All participants were aged between 10 and 20 years old (M = 13.09; SD = 2.47) and belonged to 13 different schools in Southwest Spain. They completed the cooperative learning questionnaire (CLQ), referring to the PE classes, in addition to the emotional intelligence questionnaire in physical education (EIQPE). Positive and significant associations were found between CL and EI in all school stages. In addition, moderately strong associations were uncovered between CL and the different dimensions of EI: emotional recognition, emotional control and regulation and emotional empathy. Participants belonging to classrooms with larger cooperation indices presented higher levels of EI. Results also highlighted a greater use of CL in PE classes during the PED stage in comparison to the SED and BA stages. These outcomes are discussed in light of the existing literature and methodological implications are derived for teaching PE. The use of CL in PE is recommended because of its positive contribution to the affective domain through IE. This will be especially important during the SED and BA stages, where lower rates of CL were observed.

## 1. Introduction

One of the most important decisions to make when planning physical education classes has to do with the type of methodology to be used. Teachers and lecturers must define the characteristics of their teaching intervention based on the educational objectives to be pursued. A broad array of methodological options exist that have evolved from styles based on direct instruction to frameworks that place the learner at the center of the teaching-learning process [1,2]. Within this range of methodological options, the pedagogical model of cooperative learning (CL) can be found where the focus of attention is the student [3].

CL initially emerged as a response to the low levels of interaction observed in the classrooms. One of its main goals is to facilitate relatedness between individuals in the teaching-learning process [4]. From this didactic standpoint, students learn with, by and for other students via situations characterized by promotive positive interaction and interdependence. In this context, teachers and students act as co-learners [5,6]. Correct CL implementation in the classroom is not easy and requires the presence of five key aspects: positive interdependence, promotive interaction, individual accountability, group processing and social skills (for a review, [7]). Two additional elements could also be added: equal participation and simultaneous interaction [8]. Thus, the characteristics that are inherent to CL confer this methodological tool a high educational value. This links it directly to the scripted curriculum and the function that should be played by the school in regards to students’ basic training [9].

CL implementation has been successfully conducted in physical education, and a number of meta-analyses showed strong evidence of its effectiveness [10,11,12,13,14,15]. Nowadays, it is widely accepted that engaging in group activities in physical education classes is beneficial. However, it looks like this idea is not being implemented effectively. Working in a group is not the same as proposing team activities where the five keys elements that make up CL can converge. This fact was confirmed by the two most recent literature reviews conducted on CL in physical education [16,17].

The systematic review conducted by [17] analyzed 27 different studies, but only six confirmed the fulfilment of the five structural elements essential in CL. On the other hand, the systematic review by [16], which also filtered all identified studies according to this same premise, uncovered a total of 15 eligible documents for review. Thus, we can confirm that 21 CL studies related to CL in physical education are currently available. Although the volume of analyzed studies is not excessively high due to the use of demanding and relevant inclusion criteria, clear evidence was found on the effectiveness of CL to improve behavioral outcomes related to physical education. Both systematic reviews reported effects on the cognitive, physical, social, or affective domains linked to learning. Thus, the positive outcomes of CL implementation have been verified in social/motor skills [18,19], social skills [20,21,22], motor/physical skills [23,24], cognitive skills [25,26] and affective learning [27].

Nevertheless, it was observed that a lower number of studies have been conducted on the affective and emotional dimensions of CL. Despite the importance of emotions and the incredibly relevant role they play in learning, the connection between CL and emotional intelligence (EI) has received scant attention by researchers [16,17]. Introduced by [28], the term emotional intelligence refers to the capacity to learn and manage emotions in order to channel them in a positive way. Individuals can make emotions work for them and not against them. Scientific literature outlines three main models of emotional intelligence: the ability model or four-branch model developed by [28], the model of emotional competencies described by [29,30] and the model of emotional and social intelligence illustrated by [31]. In all these models, elements such as self-regulation, self-awareness, empathy and social skills have been linked with satisfactory collaborative working [32]. Evidence exists of the important benefits that can be provided by EI in the educational context, such as subjective well-being [33] and academic performance [34]. Furthermore, a meta-analysis carried out by [35] highlighted that EI is not stable, but that it can be modified and improved. Recent research has emphasized the contribution made by physical activity and the positive role it can play on students’ IE [36,37,38], specifically, through engagement in organized sport [39] and body image satisfaction [40]. PE classes can promote the development of students’ EI [41], and CL could be a useful methodological tool to do it because of the structural elements that it includes. Previously reported studies in educational contexts have shown that adequate EI in group members can lead them to develop greater levels of positive interdependence and promotive interaction [32]. As these are fundamental elements of CL, this finding gives a clear indication of a relationship between both frameworks.

Thus, in response to the needs indicated by the literature reviewed above, the first objective of the present research was to examine the connections between students’ perceptions of CL and EI in physical education classes. Complementary; the second aim of the present study was to explore and compare levels of CL in primary education (PED), secondary education (SED) and baccalaureate (BA). This is important since this second issue was also raised in previous studies.

## 2. Method

### 2.1. Design

The study followed an ex-post facto, cross-sectional research design (quantitative methodology).

### 2.2. Participants

A total of 1332 students (682 males and 650 females) enrolled in PED (*n* = 586), SE (*n* = 551) and BA (195) agreed to participate in the study. Participants had self-reported ages between 10 and 20 years (M = 13.09; SD = 2.47). The sample was selected through multi-stage conglomerate sampling and random selection of 13 schools administering various branches of the following courses: years 6 and 7 of PED; years 8, 9, 11 and 11 of SE and years 12 and 13 of BA. The 13 randomly selected schools were located in Southwest Spain (the area were the research team works). All schools were public, situated in urban, lower-middle socioeconomic level neighborhoods.

### 2.3. Instruments

Cooperative Learning Questionnaire (CLQ) [42]. This scale measures the essential components of CL in the classroom. It was used in the present research adding the stem: “In physical education classes …”. It includes 20 items grouped in five dimensions (four items each): social skills (e.g., “We reach an agreement when presented with different or conflictive opinions”), group processing (e.g., “We discuss ideas between all members of the group”), positive interdependence (e.g., “The better each group member completes their task, the better the result obtained by the group”), promotive interaction (e.g., “We relate with each other in order to carry out activities”), and individual accountability (e.g., “Each group member must do their part of group work in order to complete the task”). A 5-point Likert scale was used to rate responses, ranging from one (totally disagree) to five (totally agree). The CLQ also produces a global cooperation factor, obtained of the mean scores of the five sub-scales. In the original validation study, the scale presented acceptable Cronbach alphas scores between 0.72 and 0.89. Good fit was also observed through confirmatory factor analysis: S-Bχ^2^ (160) = 2574.51, *p* < 0.001; CFI = 0.953; RMSEA (90% CI) = 0.037 (0.035–0.038), SRMR = 0.02. A similar result was obtained for the global cooperation factor: S-Bχ^2^ (165) = 3134.01, *p* < 0.001; CFI = 0.942; RMSEA (90% CI) = 0.040 (0.039–0.041), SRMR = 0.032.

Emotional Intelligence Questionnaire in Physical Education (EIQPE) [43]. This scale includes 22 items and 3 factors: emotional recognition (e.g., “It is easy for me to recognize my emotions during activities”), emotional control and regulation (e.g., “I am able to remain calm even when situations arise to challenge me”), and emotional empathy (e.g., “I easily understand how my classmates feel”). All items began with the stem: “In my physical education classes …”. Emotional recognition includes eight items, while emotional control and regulation, and emotional empathy include seven items. A 5-point Likert scale was used to rate responses, ranging from one (totally disagree) to five (totally agree). In the original validation study, the scale showed acceptable Cronbach alpha scores between 0.88 and 0.90. Confirmatory factor analysis presented good fit: S-Bχ^2^ (206) = 756.67, *p* < 0.001, CFI = 0.96, RMSEA = 0.040, 90% CI (0.037, 0.043), SRMR = 0.03.

### 2.4. Procedure

In order to conduct this cross-sectional study, data collection lasted three months. Questionnaires were completed in quiet classrooms at the 13 schools. Parents and/or legal guardians were fully informed of the aims of the study and provided informed written consent for the students to voluntarily participate. The study was conducted in accordance with ethical principles regulating human research [44]. The Bioethics and Biosafety Committee of the University of Extremadura gave approval for the study to be conducted (N°: 0063/2018). All collected data were handled anonymous and confidentially, in compliance with the guidelines of the American Psychological Association.

### 2.5. Data Analysis

Firstly, the assumptions of normality (Kolmogorov–Smirnov test), randomization (Rachas test) and homoscedasticity (Levene test) were fulfilled. Results showed significance values of *p* > 0.05 in all variables, justifying the use of parametric tests. With the aim of analyzing possible associations between CL and EI, bivariate correlations were performed between all variables. Odds Ratio (OR) tests were also used to estimate the association between CL and EI. Finally, MANOVA and ANOVA tests were conducted to compare CL levels between the different educational stages (PED, SED, BA).

## 3. Results

First, internal reliability coefficients, descriptive statistics, and bivariate correlations between variables are presented according to the educational stage (Table 1 and Table 2). All variables showed acceptable internal consistency scores (α > 0.70). Highest CL and EI scores were obtained in PED. A trend was observed towards decreasing mean CL scores as the educational stage increased. The same pattern was observed for EI, although less accentuated. Positive and significant associations were found between the global cooperation factor and EI in all educational stages, with BA showing the weakest correlations.

OR were also calculated to examine associations between the degree of cooperation perceived in the classroom and the EI level manifested. For this analysis, the global cooperation factor (independent variable) was dichotomized using the 50th percentile as criteria to determine high and low cut-points. The dependent variables related to EI (recognition, regulation and control, and empathy) were also dichotomized according to this same criterion (high, low). Recounts of the number of participants classified and percentage distribution of the resulting sample can be observed in Table 3.

Probabilities were calculated taking participants belonging to the high CL group as a reference relative to those in the low CL group. Next, the risk of occurrence (prognosis) or presenting high EI was estimated. Thus, belonging to the high CL group was positively and significantly associated (χ^2^ = 137.34, *p* < 0.001) with high levels of emotional recognition (OR = 3.79, 95% CI = 3.02–4.75). This relationship presented a moderate effect size, according to the test of equivalence established via Cohen’s *d* [45]. In the same trend, a positive and significant association was found (χ^2^ = 184.41, *p* < 0.001) with regards to emotional control and regulation (OR = 4.78, 95% CI = 3.79–6.02); this time with a large effect size. Finally, regarding emotional empathy (χ^2^ = 138.99, *p* < 0.001), a moderate association was observed (OR = 3.82, 95% CI = 3.04–4.79). Thus, individuals belonging to the high CL group were 3.79 times more likely to have high emotional recognition compared to the low CL group. Along the same line, when comparing high and low CL groups, the likelihood of having high emotional control and regulation and high emotional empathy was 4.78 and 3.82 times greater, respectively.

In order to assess the role of CL level across the educational stages in relation to the EI-related variables, multivariate comparisons were conducted using the average scores obtained for emotional recognition, emotional control and regulation and emotional empathy. Furthermore, analyses were conducted based on CL level, educational stage and the interaction of both variables (Table 4). Multi-factorial analysis (MANOVA) revealed significant main effects for CL level (Wilks λ = 0.863, F (3, 1324) = 70.268, *p* < 0.001, η = 0.137), educational stage (Wilks λ = 0.964, F(6, 2648) = 8.114, *p* < 0.001, η = 0.018) and the CL level/educational stage interaction (Wilks λ = 0.989, F(6, 2648) = 2.414, *p* = 0.025, η = 0.005).

Regarding emotional recognition, univariate comparisons indicated that students belonging to classrooms with high CL levels obtained higher scores than students with low CL (F(1, 1326) = 115.80, *p* < 0.001, η = 0.080). Significant differences were not found between educational stages. With regards to emotional control and regulation, univariate comparisons indicated that students who belonged to classrooms with a higher CL level obtained higher scores than students in low CL classrooms (F(1.1326) = 121.79, *p* < 0.001, η = 0.084). Higher scores were also obtained in PED students, relative to SED and BA students (F(2.1326) = 19.54, *p* < 0.001, η = 0.029). The Bonferroni test showed significant differences between PED and SED (I-J = 0.231, *p* < 0.001) and BA (I-J = 0.445, *p* < 0.001), with SED students also reporting significantly different scores to BA students (I-J = 0.214, *p* < 0.001). Finally, a significant interaction was observed between CL level and school stage (F(2.1326) = 6.317, *p* = 0.002, η = 0.009). Finally, regarding emotional empathy, univariate comparisons indicated that students belonging to classrooms characterized by high CL levels obtained higher scores than those attending classrooms with low CL (F(1.1326) = 124.52, *p* < 0.001, η = 0.086). No significant differences emerged between the different educational stages nor in relation to the CL level/educational stage interaction.

With the aim of comparing the influence of CL perceptions on the different dimensions of EI, three groups were generated using terciles of high, medium and low cut-points. ANOVA analysis found significant differences between the three studied groups, with large effect sizes (Table 5). The highest scores for all EI dimensions corresponded to the high cooperation group, with the moderately cooperative group having the next EI scores.

Lastly, Table 6 shows the differences between CL dimensions according to the educational stage. All CL dimensions, including global CL, were significantly different. Highest CL values appeared in PED, with the dimensions of interpersonal skills and global CL standing out with medium effect sizes. Lowest scores for CL were recorded in BA, with SED displaying intermediate values.

## 4. Discussion

The present research responded to the claim to conduct studies to assess the links between CL and the affective domain in physical education and to examine these connections in different educational stages. Therefore, this study had two complementary aims: (a) to examine the associations between CL and EI in physical education and (b) to compare the use of CL in PED, SED and BA. Results showed positive and significant links between both variables (CL, EI) in all educational stages (PED, SED and BA). Further, OR testing confirmed these positive association, revealing 3-to-5-times greater probability of students in high CL classrooms of having high EI. These findings suggest that CL may contribute to the development of students’ EI in physical education. In addition, a global cooperation index was found a sensitive variable to discriminate levels of EI.

With regards to the first objective, previously reported studies on group work indicated the positive role that engaging in greater amounts of physical activity [37] or organized sport [39] plays on individuals’ EI development, but no research has assessed the role that CL in physical education may play in this development. The present study reiterates the importance of using CL as an instructional framework in physical education to promote EI in PED, SED and BA [46]. Fundamental elements of CL such as individual accountability and group processing may help develop elements of EI such as self-regulation and empathy [32]. Results of the present study strengthen this idea since high levels of CL were linked to higher levels of EI. On the other hand, positive social skill development has been highlighted as a fundamental element of both approaches [7,29], with those skills strengthening the connections between both. Furthermore, [47] found that effects of CL on student empathy were mediated by peer relationships. In other words, an element as important to CL as working in small heterogeneous groups may promote the development of empathy, being this one of the most fundamental elements of EI. Results of the present study corroborate this idea because students with higher CL also reported higher EI. In addition, previously conducted studies in general educational contexts have linked the connections between CL and EI to the successful delivery of anti-bullying programs [48]. Finally, high CL levels alongside high levels of self-regulation, which is another fundamental element of EI, were correlated with high levels of academic self-efficacy in a broad student sample [49]. This emphasizes the value of the positive impact of this connection, since this same relationship also emerged in the present study. Unfortunately, no similar studies have been conducted in physical education to verify the links found here.

The second objective of the present research was to explore and compare the use of CL in the different educational stages: SED, PED and BA. Results indicated that as the educational stage moved up, students perceived CL to be promoted to a lesser extent by their teachers. In a previous study [42], trends evolved inversely: Older students recorded higher scores in all fundamental components of CL. Differences in teacher training and geographical location of the schools could explain the different results. Results from the present study also showed a progressive decrease in scores relating to EI as the educational school stage advanced. The significant decrease observed in the use of CL in physical education when comparing PED with SED and BA may be caused by the training differences between teachers. Another important influence could be the methodological decisions made by the teachers when designing the teaching-learning process [50,51]. SED and BA are more disciplinary and segmented educational stages, compared to the holistic nature of PED. In this framework, CL receives less attention from SED and BA teachers who opt for more traditional instructional methods centered on theory and individual performance. Another potential explanation for this significant decline is that, traditionally, physical education delivered in SED and BA focuses on performance (performance-oriented), and competition is promoted consciously or unconsciously, whilst physical education in PED is centered on participation (participation-oriented) and play, with cooperation being more openly promoted [52,53]. It is suggested that teachers in SED and BA should promote CL in their physical education classes, just as PED teachers do, to favor students’ EI and other learning outcomes [46,54].

The present research has a number of important strengths. It is the first study to examine the associations between CL and EI, making use of specific instruments to measure these variables in physical education. This bestows the present research with strong situational and ecological validity. Further, the approach selected to understand the subjective perceptions provided by students on the use of CL by their teachers was based on five dimensions, which were embedded into the methodological structure on the study. The present study also fills a hole in the existing literature, which lacked comparative studies of CL use in different educational stages.

### Limitations and Future Directions

One of the main limitations of the present work is the cross-sectional design, so causal relationships cannot be established. Further, validity of the data may be questioned, to a certain extent, given that the assessment of CL used by the teachers was limited to the students’ subjective perceptions. Only self-reported (self-administered) data obtained through a questionnaire was available. As no other methods were used, no comparisons can be made to provide greater measurement validity. On the other hand, the particular context of the sample should lead us to consider the results with caution. Nonetheless, future research lines have been opened on CL and the affective domain with the aim of advancing in their understanding. On the other hand, [55] have proposed practical intervention strategies to promote affective learning through two cooperative learning structures: student team assessment divisions (STAD) and jigsaw classroom. Future research should implement these structures to better understand the effectiveness of quasi-experimental interventions in physical education classes. Another important point to consider is that recent research [56], framed around a novel gamification approach that includes CL, also pointed to an incipient setting to promote affective learning. The cited study found positive effects on the students’ intrinsic motivation and enjoyment [57]. Thus, the present research offers a new contribution, which expands the possibilities to put cooperative mechanisms into action and enable students to experience positive emotions to develop EI.

## 5. Conclusions

The present research offers two main implications for teaching practice in physical education. (i) It is suggested that teachers should be trained in CL and its implementation, because of the positive association observed between CL and the affective domain (EI), in PED, SED and BA students. (ii) Teachers should use CL basic principles in their classes, especially during SED and BA, when students perceived fewer structural elements, which are cooperative in nature, and reported lower EI levels relative to PED.

## Figures and Tables

**Table 1:** Alphas, means, standard deviations and bivariate correlations of the observed variables in primary school (below) and secondary school (above) students.

Variables	Cooperative Learning						Emotional Intelligence		
	1	2	3	4	5	6	7	8	9
α	0.76	0.77	0.73	0.78	0.79	0.87	0.84	0.82	0.83
M	3.29	3.49	3.87	3.94	4.20	3.76	4.01	3.55	3.58
SD	0.90	0.89	0.70	0.74	0.72	0.61	0.66	0.74	0.68
1.Interpersonal skills	-	0.732 **	0.496 **	0.432 **	0.293 **	0.795 **	0.270 **	0.409 **	0.351 **
2. Group processing	0.621 **	-	0.529 **	0.494 **	0.308 **	0.820 **	0.309 **	0.421 **	0.346 **
3. Positive interdependence	0.421 **	0.440 **	-	0.574 **	0.557 **	0.800 **	0.364 **	0.308 **	0.328 **
4. Promotive interaction	0.474 **	0.512 **	0.547 **	-	0.520 **	0.767 **	0.325 **	0.335 **	0.298 **
5. Individual Accountability	0.335 **	0.379 **	0.507 **	0.528 **	-	0.666 **	0.435 **	0.276 **	0.333 **
6. Global cooperation factor	0.769 **	0.791 **	0.756 **	0.793 **	0.699 **	-	0.435 **	0.460 **	0.430 **
7. Emotional recognition	0.365 **	0.345 **	0.349 **	0.380 **	0.349 **	0.468 **	-	0.479 **	0.501 **
8. Emotional control regulation	0.342 **	0.338 **	0.260 **	0.346 **	0.195 **	0.392 **	0.490 **	-	0.475 **
9. Emotional empathy	0.360 **	0.372 **	0.309 **	0.364 **	0.228 **	0.432 **	0.377 **	0.447 **	-
α	0.75	0.76	0.72	0.73	0.78	0.86	0.86	0.82	0.82
M	3.74	3.79	4.14	4.12	4.45	4.05	4.10	3.78	3.65
SD	0.78	0.77	0.69	0.67	0.64	0.54	0.61	0.64	0.73

** *p* < 0.01.

**Table 2:** Alphas, means, standard deviations and bivariate correlations of the observed variables in students across all school stages (below) and those undertaking baccalaureate study (above).

Variables	Cooperative Learning						Emotional Intelligence		
	1	2	3	4	5	6	7	8	9
α	0.73	0.74	0.73	0.75	0.79	0.85	0.84	0.83	0.82
M	2.92	3.25	3.87	3.96	4.29	3.66	3.97	3.34	3.52
SD	0.79	0.83	0.71	0.72	0.69	0.60	0.63	0.69	0.66
1. Interpersonal skills	-	0.753 **	0.515 **	0.592 **	0.414 **	0.829 **	0.117	0.155 *	0.233 **
2. Group processing	0.712 **	-	0.561 **	0.661 **	0.459 **	0.869 **	0.145 *	0.079	0.214 **
3. Positive interdependence	0.490 **	0.515 **	-	0.573 **	0.532 **	0.784 **	0.175 *	0.092	0.268 **
4. Promotive interaction	0.476 **	0.532 **	0.571 **	-	0.499 **	0.826 **	0.246 **	0.124	0.295 **
5. Individual accountability	0.347 **	0.373 **	0.546 **	0.529 **	-	0.704 **	0.266 **	0.135	0.244 **
6. Global cooperation factor	0.804 **	0.826 **	0.787 **	0.783 **	0.690 **	-	0.230 **	0.145 *	0.310 **
7. Emotional recognition	0.295 **	0.308 **	0.337 **	0.341 **	0.381 **	0.421 **	-	0.350 **	0.326 **
8. Emotional control regulation	0.395 **	0.374 **	0.283 **	0.321 **	0.244 *	0.421 **	0.468 **	-	0.294 **
9. Emotional empathy	0.340 **	0.342 **	0.315 **	0.329 **	0.278 **	0.413 **	0.424 **	0.439 **	-
α	0.74	0.76	0.74	0.77	0.80	0.86	0.85	0.82	0.83
M	3.43	3.59	3.99	4.03	4.32	3.87	4.05	3.62	3.60
SD	0.89	0.85	0.71	0.71	0.69	0.60	0.64	0.71	0.70

* *p* < 0.05; ** *p* < 0.01.

**Table 3:** Recount of classified participants and percentage sample distribution.

Variables			Emotional Recognition			Emotional Control and Regulation			Emotional Empathy		
			High	Low	Total	High	Low	Total	High	Low	Total
Cooperative Learning	High	*N*	452	225	677	473	204	677	443	234	677
		%	66.6	34.5	50.8	68.9	31.6	50.8	67.1	34.8	50.8
	Low	*N*	227	428	655	214	441	655	217	438	655
		%	33.4	65.5	49.2	31.1	68.4	49.2	32.9	65.2	49.2
	Total	*N*	679	653	1332	687	645	1332	660	672	1332
		%	100	100	100	100	100	100	100	100	100

**Table 4:** Means and standard deviations for emotional intelligence based on cooperative learning level and educational stage.

Variables	CL Level	Primary Education		Secondary Education		Baccalaureate	
		M	SD	M	SD	M	SD
Emotional Recognition	High	4.26	0.53	4.32	0.49	4.15	0.60
	Low	3.82	0.65	3.79	0.68	3.87	0.63
	Total	4.09	0.61	4.01	0.66	3.97	0.63
Emotional Control and Regulation	High	3.92	0.60	3.92	0.60	3.52	0.75
	Low	3.50	0.61	3.28	0.72	3.24	0.64
	Total	3.78	0.64	3.55	0.74	3.33	0.69
Emotional Empathy	High	3.82	0.69	3.90	0.56	3.74	0.58
	Low	3.34	0.70	3.34	0.66	3.39	0.68
	Total	3.65	0.73	3.58	0.68	3.51	0.66

Note: CL = Cooperative Learning.

**Table 5:** ANOVA analysis of differences in emotional intelligence dimensions according to cooperation levels.

Variables	High Cooperation ^1^ (*N* = 431)	Medium Cooperation ^2^ (*N* = 462)	Low Cooperation ^3^ (*N* = 439)	*F*	*p*	*η* ^2^
Emotional recognition	4.35 ± 0.51 ^2,3^	4.03 ± 0.53 ^1,3^	3.76 ± 0.71 ^1,2^	109.875	0.000	0.14
Emotional control and regulation	3.96 ± 0.65 ^2,3^	3.62 ± 0.63 ^1,3^	3.29 ± 0.70 ^1,2^	110.886	0.000	0.14
Emotional empathy	3.94 ± 0.64 ^2,3^	3.59 ± 0.59 ^1,3^	3.28 ± 0.71 ^1,2^	114.935	0.000	0.15

Note: Bonferroni post hoc test (*p* < 0.01); superscripts indicate differences between cooperation levels. *η*^2^: Effect size.

**Table 6:** ANOVA analysis of differences in the cooperative learning dimensions according to educational stage.

Variables	Primary Education ^1^ (*N* = 586)	Secondary Education ^2^ (*N* = 551)	Baccalaureate ^3^ (*N* = 195)	*F*	*p*	*η* ^2^
Interpersonal skills	3.74 ± 0.78 ^2,3^	3.29 ± 0.90 ^1,3^	2.92 ± 0.79 ^1, 2^	83.865	0.000	0.11
Group processing	3.79 ± 0.77 ^2,3^	3.49 ± 0.89 ^1,3^	3.25 ± 0.83 ^1, 2^	37.214	0.000	0.05
Positive interdependence	4.14 ± 0.69 ^2,3^	3.87 ± 0.70 ^1^	3.87 ± 0.71 ^1^	25.072	0.000	0.04
Promotive interaction	4.12 ± 0.67 ^2,3^	3.94 ± 0.74 ^1^	3.96 ± 0.72 ^1^	9.756	0.000	0.01
Individual Accountability	4.45 ± 0.64 ^2,3^	4.20 ± 0.72 ^1^	4.29 ± 0.69 ^1^	20.260	0.000	0.03
Global cooperation factor	4.05 ± 0.54 ^2,3^	3.76 ± 0.61 ^1^	3.66 ± 0.60 ^1^	50.907	0.000	0.07

Note: Bonferroni post hoc test (*p* < 0.01); superscripts indicate differences between school stages. *η*^2^: Effect size.
